# mTOR Inhibitors Control the Growth of EGFR Mutant Lung Cancer Even after Acquiring Resistance by HGF

**DOI:** 10.1371/journal.pone.0062104

**Published:** 2013-05-14

**Authors:** Daisuke Ishikawa, Shinji Takeuchi, Takayuki Nakagawa, Takako Sano, Junya Nakade, Shigeki Nanjo, Tadaaki Yamada, Hiromichi Ebi, Lu Zhao, Kazuo Yasumoto, Takahiro Nakamura, Kunio Matsumoto, Hiroshi Kagamu, Hirohisa Yoshizawa, Seiji Yano

**Affiliations:** 1 Division of Medical Oncology, Cancer Research Institute, Kanazawa University, Kanazawa, Japan; 2 Division of Tumor Dynamics and Regulation, Cancer Research Institute, Kanazawa University, Kanazawa, Japan; 3 Department of Medicine (II), Niigata University Medical and Dental Hospital, Niigata City, Japan; Seoul National University, Korea

## Abstract

Resistance to epidermal growth factor receptor tyrosine kinase inhibitors (EGFR-TKIs), gefitinib and erlotinib, is a critical problem in the treatment of *EGFR* mutant lung cancer. Several mechanisms, including bypass signaling by hepatocyte growth factor (HGF)-triggered Met activation, are implicated as mediators of resistance. The mammalian target of rapamycin (mTOR), is a downstream conduit of EGFR and MET signaling, and is thus considered a therapeutically attractive target in the treatment of various types of cancers. The purpose of this study was to examine whether 2 clinically approved mTOR inhibitors, temsirolimus and everolimus, overcome HGF-dependent resistance to EGFR-TKIs in *EGFR* mutant lung cancer cells. Both temsirolimus and everolimus inhibited the phosphorylation of p70S6K and 4E-BP1, which are downstream targets of the mTOR pathway, and reduced the viability of *EGFR* mutant lung cancer cells, PC-9, and HCC827, even in the presence of HGF *in vitro*. In a xenograft model, temsirolimus suppressed the growth of PC-9 cells overexpressing the *HGF*-gene; this was associated with suppression of the mTOR signaling pathway and tumor angiogenesis. In contrast, erlotinib did not suppress this signaling pathway or tumor growth. Multiple mechanisms, including the inhibition of vascular endothelial growth factor production by tumor cells and suppression of endothelial cell viability, contribute to the anti-angiogenic effect of temsirolimus. These findings indicate that mTOR inhibitors may be useful for controlling HGF-triggered EGFR-TKI resistance in *EGFR* mutant lung cancer, and they provide the rationale for clinical trials of mTOR inhibitors in patients stratified by *EGFR* mutation and HGF expression status.

## Introduction

Lung cancer is the leading cause of malignancy-related death worldwide, and more than 80% of cases are classified as non-small cell lung cancer (NSCLC). Epidermal growth factor receptor (EGFR) activating mutations, such as exon 19 deletion and exon 21 L858R point mutation, are found in a population of NSCLC, and are associated with a clinical response to the EGFR tyrosine kinase inhibitors (EGF-TKIs), gefitinib and erlotinib [1]–[3]. However, almost all responders acquire resistance and develop recurrence after varying periods of time (acquired resistance) [4]. In addition, 20–30% of the patients show unfavorable responses, although their tumors have target mutations (intrinsic resistance) [5]. Many studies have been performed in order to delineate strategies that may overcome acquired and intrinsic resistance. These studies have identified several mechanisms of acquired resistance, including *EGFR* T790M mutation [6], [7], *MET* amplification [8], [9], hepatocyte growth factor (HGF) overexpression [10], loss of PTEN [11], transformation to a small cell lung cancer (SCLC) phenotype [12]–[14], epithelial-to-mesenchymal transition (EMT) [15]–[17], activation of the NFkB pathway [18], alteration of microRNA [19], and Gas6-Axl axis activation [20]. The complexity of NSCLC is reflected by the co-occurrence of various combinations of these resistance mechanisms in different individuals. We previously discovered that HGF triggers EGFR-TKI resistance by activating the MET/PI3K/AKT axis [10]. Furthermore, we showed that HGF overexpression is present in tumors from Japanese patients with acquired and intrinsic tumor resistance to EGFR-TKI at frequencies of about 60% and 30%, respectively [21]. This indicates that HGF is an ideal target for overcoming EGFR-TKI resistance in *EGFR* mutant lung cancer patients. To overcome HGF-triggered resistance, 2 signals from EGFR and HGF-MET should be blocked simultaneously. We already reported that HGF-dependent resistance can be controlled by an anti-HGF neutralizing antibody [22], the HGF antagonist NK4 [22], MET-TKI [23]–[25], and phosphatidylinositol 3-kinase (PI3K) inhibitors [26] in combination with EGFR-TKI. However, these inhibitors are not clinically approved and therefore cannot be used for treatment of cancer patients.

The mammalian target of rapamycin (mTOR), a serine/threonine kinase, is a downstream target of the PI3K and AKT pathways, and it plays a critical role in cell survival and proliferation [27]–[29]. Activation of PI3K/AKT and subsequent phosphorylation of mTOR initiates the phosphorylation of important downstream targets, including ribosomal p70S6 serine/threonine kinase (S6K1) and eukaryotic initiation factor (EIF)-4E binding protein (4E-BP1), resulting in an increase in mRNA translation and cap-dependent protein synthesis, respectively. Thus, mTOR kinase is a key node of the PI3K/AKT signaling pathway [27]–[30]. To date, several mTOR inhibitor rapamycin analogs have been developed, including temsirolimus and everolimus, which have been used to treat renal cell carcinomas and pancreatic neuroendocrine tumors. Rapamycin and its analogs bind FK506-binding protein-12 (FKBP12) and interact with mTOR, inhibiting its kinase activities and halting the translation of proteins critical for cell proliferation and survival.

Because mTOR is downstream of both EGFR and MET, we hypothesized that mTOR inhibition, even as a monotherapy agent, may block EGFR- and MET-mediated signaling simultaneously and overcome HGF-triggered EGFR-TKI resistance. In the present study, we examined whether the clinically approved mTOR inhibitors, temsirolimus and everolimus, circumvent HGF-triggered EGFR-TKI resistance in *EGFR* mutant lung cancer cells using *in vitro* and *in vivo* models, and assessed underlying mechanisms.

## Materials and Methods

### Cell cultures and reagents

*EGFR* mutant human lung adenocarcinoma cell lines PC-9 (del E746_A750) and HCC827, with deletions in *EGFR* exon 19, were purchased from Immuno-Biological Laboratories Co. (Takasaki, Gunma, Japan) and the American Type Culture Collection (Manassas, VA), respectively. Human *HGF*-gene transfectants (PC-9/HGF) and vector control (PC-9/Vec) cells were established as previously described [10]. All cell lines were maintained in RPMI 1640 medium supplemented with 10% FBS and antibiotics. All cells were passaged for less than 3 months before renewal from frozen, early-passage stocks. Cells were regularly screened for mycoplasma by using MycoAlert Mycoplasma Detection Kits (Lonza, Rockland, ME). The cell lines were authenticated at the laboratory of the National Institute of Biomedical Innovation (Osaka, Japan) by short tandem repeat analysis. Erlotinib, everolimus, and temsirolimus were obtained from Selleck Chemicals (Houston, TX). Human recombinant HGF was prepared as previously described [10].

### Production of HGF and VEGF in cell culture supernatants

Cells (2×10^5^) were cultured in 2 mL medium with 10% FBS for 24 h, washed with PBS and incubated for 48 h in medium with 10% FBS. In some experiments, HGF was added to the medium. The culture media were harvested and centrifuged, and the supernatants were stored at −70°C until analysis. The concentrations of HGF and VEGF were determined by IMMUNIS HGF EIA (Institute of Immunology, Tokyo, Japan) and Quantikine VEGF ELISA (R&D Systems, Minneapolis, MN), respectively, according to the manufacturers' protocols. All samples were run in duplicate. Color intensity was measured at 450 nm using a spectrophotometric plate reader. Growth factor concentrations were determined by comparison with standard curves. The detection limits for HGF and VEGF were 100 pg/mL and 31 pg/mL, respectively.

### Cell viability assay

Cell viability was measured by the MTT dye reduction method. Tumor cells, plated at 2×10^3^/100 µL RPMI 1640 medium plus 10% FBS/well in 96-well plates, were incubated for 24 h. Then, erlotinib, temsirolimus, everolimus, and/or HGF were added to each well, and incubation was continued for an additional 72 h. Cell viability was measured with MTT solution (2 mg/mL; Sigma, St. Louis, MO), as previously described [10]. Each experiment was performed with triplicate samples.

### Antibodies and western blotting

Protein aliquots (25 µg each) were resolved by SDS polyacrylamide gel electrophoresis (Bio-Rad, Hercules, CA) and transferred to polyvinylidene difluoride membranes (Bio-Rad). After washing 4 times, the membranes were incubated with Blocking One (Nacalai Tesque, Inc., Kyoto, Japan) for 1 h at room temperature (RT) and overnight at 4°C with primary antibodies to β-actin (13E5), Met (25H2), phospho-MET (anti-p-MET, Y1234/Y1235; 3D7), p-EGFR (Y1068), AKT or p-AKT (S473), mTOR, p-mTOR (S2448), p70S6K, pp70S6K (T389), 4E-BP1, p4E-BP1(T37/46) (Cell Signaling Technology, Beverly, MA), human EGFR (1 µg/mL), human/mouse/rat ERK1/ERK2 (0.2 µg/mL), and p-ERK1/ERK2 (T202/Y204; 0.1 µg/mL) (R&D Systems). After 3 washes, the membranes were incubated for 1 h at RT with species-specific horseradish peroxidase–conjugated secondary antibodies. Immunoreactive bands were visualized with SuperSignal West Dura Extended Duration Substrate, an enhanced chemiluminescent substrate (Pierce Biotechnology, Rockford, IL).

### Xenograft studies in SCID mice

Suspensions of PC-9/Vec and PC-9/HGF cells (5×10^6^) were subcutaneously injected into the back of 5-week-old male mice with severe combined immunodeficiency (SCID) (Clea, Tokyo, Japan). After 7 days, the mice were randomized to no treatment (control group), oral erlotinib (20 mg/kg/day), or intravenous temsirolimus (50 mg/kg/week in water). Tumor size was measured twice a week, and tumor volume was calculated (mm^3^) as [(width)^2^ × length]/2. All animal experiments complied with the guidelines for the Institute for Experimental Animals, Advanced Science Research Center, Kanazawa University, Kanazawa, Japan (approval no. AP-081088).

### Histological analyses

For the detection of proliferating tumor cells (Ki-67), 5-µm-thick sections were deparaffinized in xylene, rehydrated in decreasing concentrations of ethanol, and antigen-retrieved. For detection of endothelial cells (CD31), 5-µm-thick frozen sections of xenograft tumors were fixed with cold acetone and washed with PBS. Then, endogenous peroxidase activity was blocked by incubation in 3% aqueous H_2_O_2_ for 10 min. Following treatment with 5% normal horse serum, the sections were incubated with primary antibodies to Ki-67 (MIB-1) (Dako Cytomation, Glostrup, Denmark), and mouse CD31 (MEC13.3) (BD Pharmingen, Franklin Lakes, NJ). After probing with species-specific biotinylated secondary antibodies, the sections were incubated for 30 min with avidin–biotinylated peroxidase complex (ABC) using a Vectastain ABC kit (Vector Laboratories, Burlingame, CA). The DAB (3,3′-diaminobenzidine tetrahydrochloride) Liquid System (Dako Cytomation) was used to detect immunostaining. Omission of primary antibodies served as a negative control. The 5 areas containing the highest numbers of stained cells within a section were selected for histologic quantitation by light or fluorescent microscopy at 400-fold magnification. All results were independently evaluated by 2 two investigators (D.I. and T.N.).

### Statistical analysis

Between group comparisons were assessed by two-tailed Student's *t-*tests. All analyses were performed using GraphPad Software. A *P* value<0.01 was considered statistically significant.

## Results

### mTOR inhibitors suppress viability of *EGFR* mutant lung cancer cells in the presence of HGF

**Figure 1:**
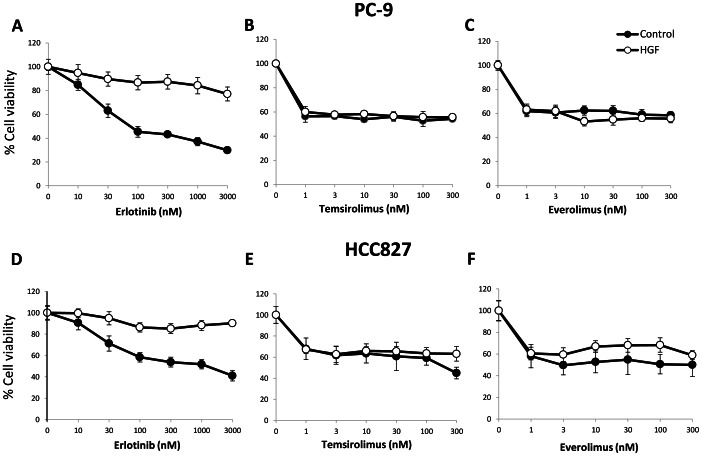
mTOR inhibitors suppressed viability of *EGFR* mutant lung cancer cells even in the presence of HGF. PC-9 or HCC827 cells were incubated with or without erlotinib, temsirolimus, or everolimus, in the presence or absence of HGF (20 ng/ml) for 72 h. Then, cell viability was determined by the MTT assay. Bars show SD. The data shown are representative of 5 independent experiments with similar results.

PC-9 and HCC827 are human lung adenocarcinoma cell lines with deletions of exon 19 in *EGFR*, resulting in constitutive activation of this protein. These cell lines were sensitive to erlotinib, whereas HGF induced erlotinib resistance (**Fig. 1**). Treatment with either mTOR inhibitor (temsirolimus or everolimus) alone discernibly suppressed the viability of these cell lines. Under these experimental conditions, HGF did not decrease the sensitivity of these cell lines to either drug. These results suggest that mTOR inhibitors may effectively control the growth of *EGFR* mutant lung cancer cells, irrespective of the presence of HGF which would induce EGFR-TKI resistance.

**Figure 2:**
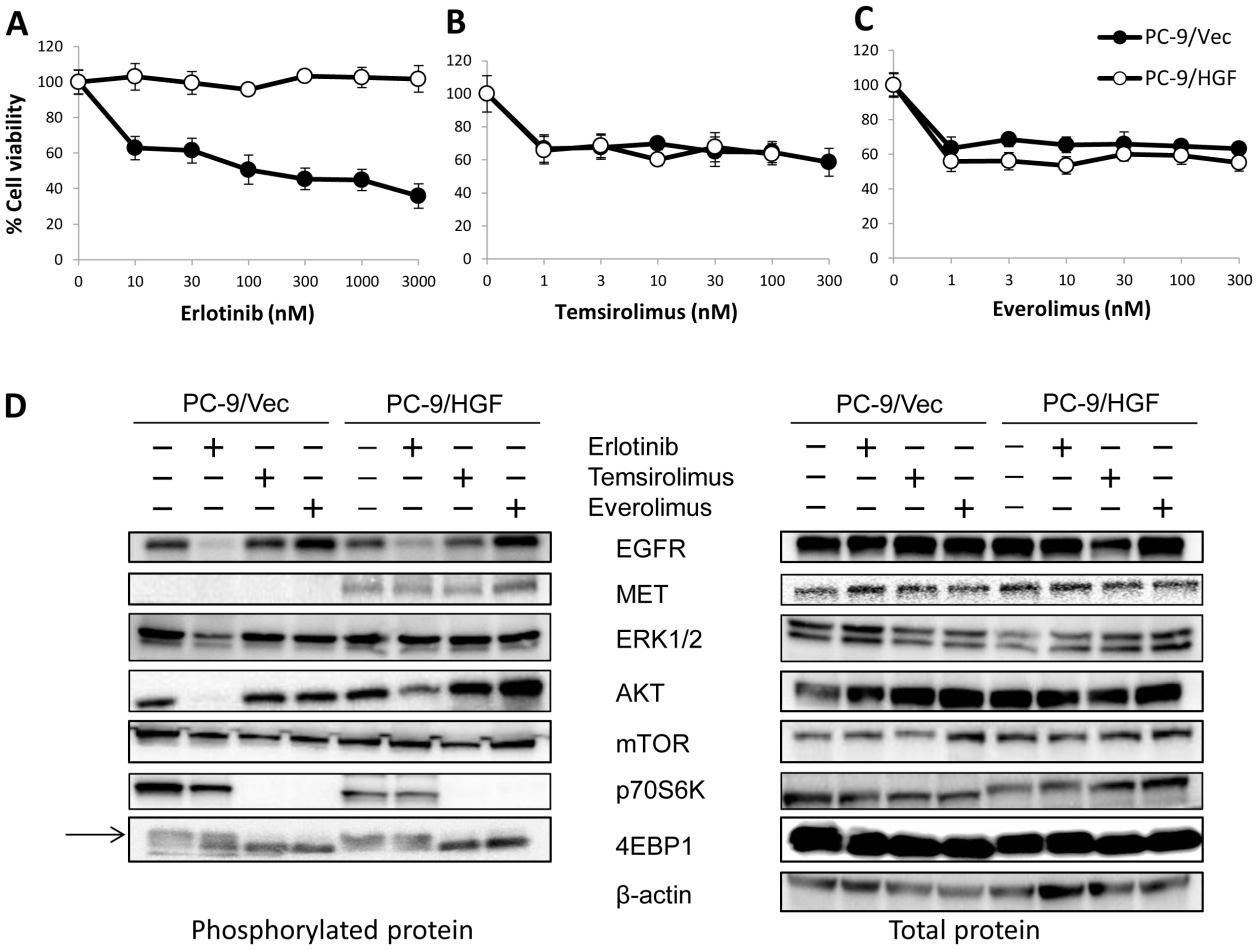
mTOR inhibitors suppressed viability of endogenously HGF-expressing *EGFR* mutant lung cancer cells via mTOR signal inhibition. PC-9/Vec and PC-9/HGF cells were incubated with erlotinib (**A**), temsirolimus (**B**), or everolimus (**C**) for 72 h. Then, cell viability was determined using the MTT assay. Bars show SD. (**D**) PC-9/Vec and PC-9/HGF cells were treated with or without erlotinib (0.3 µM), temsirolimus (0.3 µM), or everolimus (0.3 µM) for 4 h. The cell lysates were harvested and subjected to western blotting. The data shown are representative of 3 independent experiments with similar results.

Our previous study demonstrated that, in patients with NSCLC, HGF is primarily detected in cancer cells with acquired resistance to EGFR-TKIs, suggesting that the production of HGF by these cells occurs mainly via an autocrine mechanism. To further explore the effect of mTOR inhibitors on autocrine HGF production, we generated stable *HGF*-gene transfectants in PC-9 cells (PC-9/HGF). PC-9/HGF cells secreted high concentrations of HGF (28±1 ng/48 h), whereas the concentrations of HGF secreted by PC-9- and control vector-transfected PC-9/Vec cells were below the limit of detection. In addition, PC-9/HGF cells became resistant to erlotinib (**Fig. 2**). We found that temsirolimus or everolimus discernibly reduced the viability of both PC-9/Vec and PC-9/HGF cells to the same extent, whereas the combination of mTOR inhibitor plus erlotinib had no effect in PC-9/Vec cells in the presence of HGF, indicating that the mTOR inhibitors did not further sensitize these cells to erlotinib *in vitro* (**Fig. S1**). Knock down of mTOR by siRNA resulted in inhibition of viability of PC-9/Vec and PC-9/HGF cells by 30% (**Fig S2 B**), indicating that inhibited cell viability by temsirolimus even in the presence of HGF is due to mTOR inhibition. We also performed flow cytometry using Annexin V and found that temsirolimus did not induce discernible apoptosis in PC-9/Vec or PC-9/HGF cells (**Fig. S3**). These findings indicate that mTOR inhibitors, when used as single agents, suppress growth of *EGFR* mutant lung cancer cells via inhibition of proliferation rather than induction of apoptosis.

### mTOR inhibitors suppress p70S6K and 4EBP1 phosphorylation even in the presence of HGF

To explore the molecular mechanism by which mTOR inhibitors suppressed cell viability even in the presence of HGF, we examined the protein expression and phosphorylation status of EGFR, MET, and their downstream molecules (ERK1/2, PI3K/AKT, p70S6K, and 4EBP1) in PC-9/Vec and PC-9/HGF cells by western blotting (**Fig. 2D**). PC-9/Vec cells expressed EGFR and MET proteins, whereas only EGFR was discernibly phosphorylated. Erlotinib remarkably inhibited the phosphorylation of EGFR, as well as downstream ERK1/2 and AKT, but it marginally inhibited p70S6K phosphorylation. While neither temsirolimus nor everolimus affected the phosphorylation of EGFR, MET, ERK1/2 or AKT, they inhibited the phosphorylation of p70S6K and 4EBP1, the downstream molecules of mTOR.

In PC-9/HGF cells, MET was also phosphorylated, presumably due to HGF that was produced in an autocrine manner. Erlotinib inhibited EGFR phosphorylation, but it did not remarkably inhibit the phosphorylation of MET, AKT, p70S6K, or 4EBP1. While neither temsirolimus nor everolimus inhibited the phosphorylation of EGFR, MET, ERK1/2, or AKT, they markedly inhibited the phosphorylation of p70S6K and 4EBP1. These results indicate that mTOR inhibitors suppress the phosphorylation of downstream molecules of mTOR, irrespective of the presence of HGF.

### mTOR inhibition suppresses HGF-induced growth of erlotinib-resistant tumors *in vivo*

**Figure 3:**
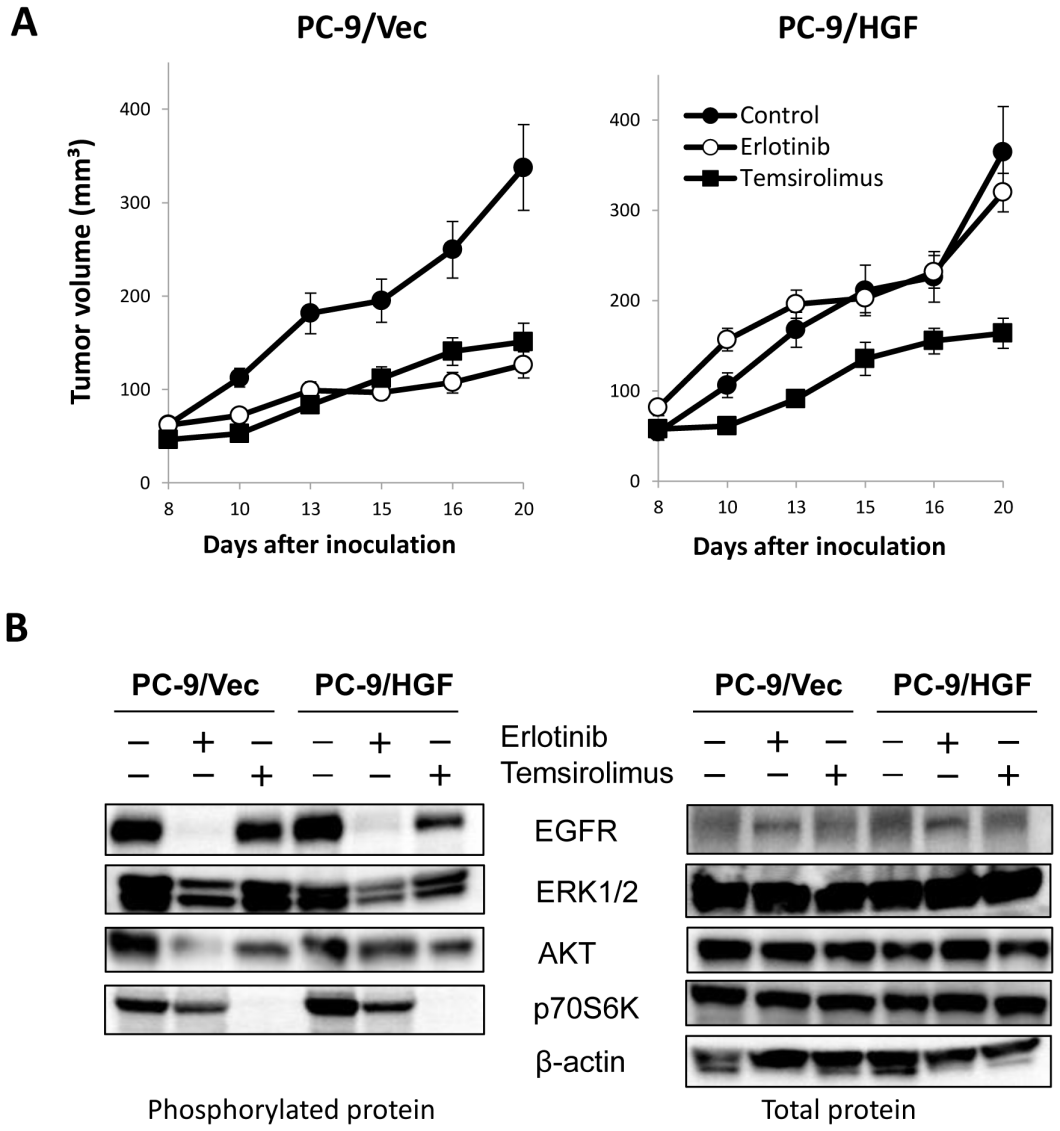
Treatment with temsirolimus inhibited the growth of erlotinib-resistant PC-9/HGF tumors in SCID mice. (**A**) SCID mice bearing PC-9/Vec or PC-9/HGF tumors were administered 25 mg/kg erlotinib once daily or 50 mg/kg temsirolimus once a week from day 8 to day 20. Tumor volume was measured using calipers on the indicated days. Mean ± SE tumor volumes are shown for groups of 5 mice. *P<0.01 (one-way ANOVA). The data shown are representative of 2 independent experiments with similar results. (**B**) SCID mice with PC-9/Vec or PC-9/HGF tumors were administered 25 mg/kg erlotinib once daily for 4 days or 50 mg/kg temsirolimus once from day 8. Four hours after final erlotinib administration, tumors were harvested, and the relative levels of proteins in the tumor lysates were determined by western blotting.

We next evaluated whether mTOR inhibitors would control the growth of *EGFR* mutant lung cancer cells with HGF-triggered EGFR-TKI-resistance *in vivo*. Oral administration of erlotinib suppressed the growth of PC-9/Vec-tumors, but not PC-9/HGF tumors, indicating resistance of PC-9/HGF tumors to erlotinib *in vivo* (**Fig. 3A**). On the other hand, intravenous administration of temsirolimus suppressed the growth of both PC-9/Vec tumors and PC-9/HGF tumors. These results indicated that mTOR inhibition may be useful for controlling the growth of HGF-induced resistant tumors *in vivo*.

We further assessed the molecular mechanisms by which temsirolimus inhibited the growth of PC-9/HGF-tumors. Western blot analyses revealed that erlotinib markedly inhibited the phosphorylation of EGFR, ERK1/2, and AKT, and slightly suppressed the phosphorylation of p70S6K in PC-9/Vec-tumors (**Fig. 3B**). While erlotinib remarkably inhibited EGFR phosphorylation, it only slightly inhibited ERK1/2 and p70S6K phosphorylation, whereas it did not inhibit AKT phosphorylation in PC-9/HGF-tumors. On the other hand, though temsirolimus did not markedly affect the phosphorylation of EGFR or ERK1/2 in both PC-9/Vec tumors and PC-9/HGF tumors, it remarkably inhibited p70S6K phosphorylation, suggesting the mode of action of this drug as a mTOR inhibitor.

Interestingly, temsirolimus slightly inhibited the Akt phosphorylation in PC-9/HGF tumors, but it increased AKT phosphorylation in PC-9/HGF cells *in vitro* condition (**Fig. 2D**). To clarify the cause of this discrepancy, we examined the time course of mTOR inhibitor treatment on AKT phosphorylation *in vivo* (**Fig S4**). We found that in accordance with the results of *in vitro* experiments, AKT phosphorylation in the tumors was increased 4 h after temsirolimus treatment. But then, the level of AKT phosphorylation was decreased and it was slightly inhibited at 96 h after the treatment, in accordance with the results shown in Fig. 3B. Therefore, the discrepancy of the results between Fig. 2D and Fig. 3B were due to the difference of the timing samples were harvested.

### mTOR inhibition suppresses angiogenesis *in vivo*

**Figure 4:**
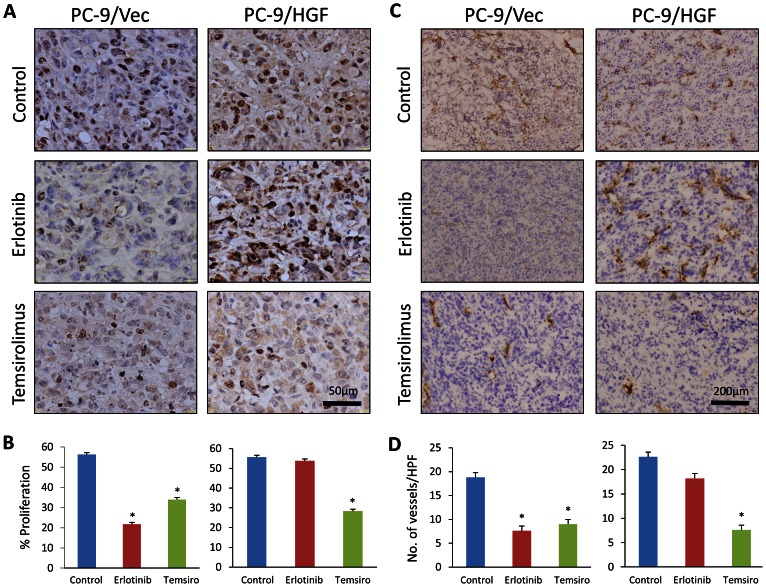
Treatment with temsirolimus inhibited tumor-cell proliferation and angiogenesis. SCID mice with PC-9/Vec or PC-9/HGF tumors were administered as described in Fig. 3B. Four hours after final administration of erlotinib, tumors were harvested, and tumor cell proliferation (**A**, **B**: Ki-67) and angiogenesis (**C, D**: CD31) were determined by immunohistochemistry. **B** and **D** show quantification of positive cells. ^*^P<0.01, (one-way ANOVA). Bars show SD.

We also examined tumor cell proliferation (Ki-67) and angiogenesis (CD31) by immunohistochemistry. Erlotinib treatment markedly inhibited tumor cell proliferation and angiogenesis in PC-9/Vec xenografts, whereas erlotinib did not affect cell proliferation or angiogenesis significantly in PC-9/HGF xenografts (**Fig. 4**). In contrast, temsirolimus markedly inhibited cell proliferation and angiogenesis in both PC-9/Vec and PC-9/HGF tumors. These findings suggest that temsirolimus inhibits the growth of PC-9/HGF tumors (at least in part) by inhibiting angiogenesis.

**Figure 5:**
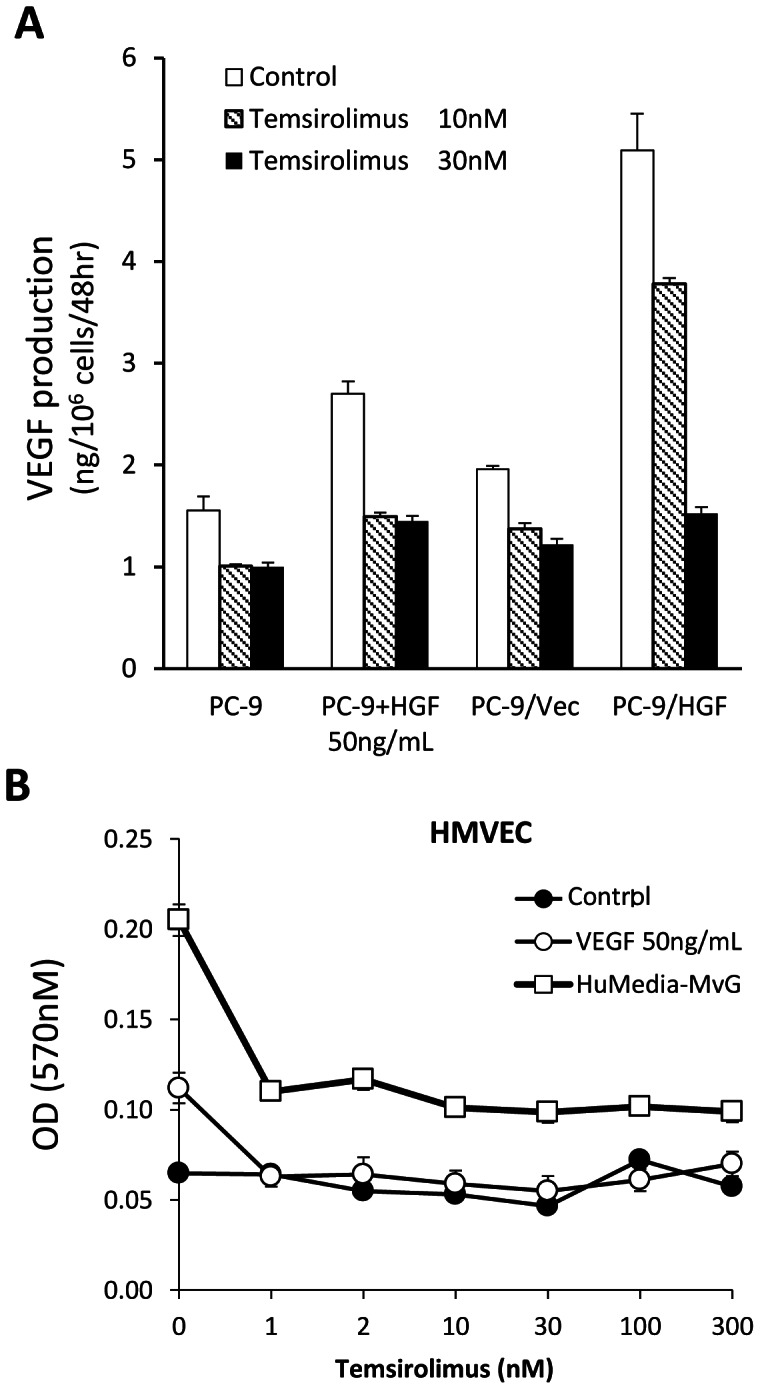
Temsirolimus inhibited VEGF production by cancer cells and endothelial proliferation. (**A**) Tumor cells were incubated with or without HGF (50 ng/ml) in the presence of different concentrations of temsirolimus for 48 h. Then, supernatants were harvested and the number of tumor cells was counted. VEGF concentration in the supernatants was determined by ELISA. VEGF levels corrected by the tumor cell number are shown. (**B**) HMVECs were incubated in RPMI-1640 medium with 10% FBS (control), RPMI-1640 medium with 10%-FBS plus VEGF, or HuMedia-MvG with different concentrations of temsirolimus for 72 h. Then, cell viability was determined using the MTT assay. Bars show SD. The data shown are 2 independent experiments with similar results.

To further explore the molecular mechanisms by which temsirolimus inhibited angiogenesis, we examined its effect on tumor cell production of VEGF, a prominent pro-angiogenic molecule. Both PC-9 and PC-9/Vec cells constitutively produced detectable levels of VEGF, which was stimulated by HGF (**Fig. 5A**). Consistent with these observations, PC-9/HGF cells produced higher levels of VEGF than PC-9 and PC-9/Vec cells. Temsirolimus suppressed VEGF production in these cancer cells in the presence or absence of HGF. Knock down of mTOR by siRNA resulted in inhibition of VEGF production by PC-9/Vec and PC-9/HGF cells, just like temsirolimus treatment (**Fig S2**). These results indicate that inhibited VEGF production by temsirolimus even in the presence of HGF is due to mTOR inhibition. We also assessed the direct effect of temsirolimus on the viability of endothelial cells *in vitro*. Temsirolimus did not inhibit constitutive viability of HMVEC cells in the medium with 10% FBS alone (**Figure 5B**). VEGF and HuMedia-MvG, which contain EGF and fibroblast growth factor-2 (FGF-2), increased the viability of HMVECs. Temsirolimus, at concentrations of 1 nM and higher, inhibited this increase in viability. These results indicate that temsirolimus inhibits angiogenesis by multiple mechanisms.

## Discussion

In the present study, we demonstrated that mTOR inhibitors suppressed the growth of *EGFR* mutant lung cancer cells, even in the presence of HGF, *in vitro* and *in vivo*. Our results also indicate that mTOR inhibitors suppress angiogenesis by inhibiting VEGF production and endothelial proliferation stimulated with various pro-angiogenic factors. These observations suggest that mTOR inhibitors, as monotherapeutic agents, are useful for controlling the progression of *EGFR* mutant lung cancer and HGF-triggered resistance to EGFR-TKIs.

HGF, also called scatter factor, is a multifunctional cytokine [31]. Recent studies showed that HGF is involved in the carcinogenesis, invasion/motility, EMT, angiogenesis, and metastasis in lung cancer, and it is therefore associated with poor prognosis of patients [32]. We previously reported that HGF induces EGFR-TKI-resistance in *EGFR* mutant lung cancer cells by restoring MET/PI3K/AKT pathway signaling [10]. HGF Overexpression is involved not only in acquired resistance but also intrinsic resistance to EGFR-TKIs [21]. In addition, HGF stimulates VEGF production by *EGFR* mutant lung cancer cells, as well as facilitates angiogenesis, thereby promoting tumor progression [24]. HGF overexpression is often co-detected with the *EGFR*-T790M gatekeeper mutation in acquired resistant tumors [9], [11], [21]. HGF induces resistance to irreversible EGFR-TKIs and mutant EGFR-selective TKIs [33], which were developed to overcome T790M-mediated EGFR-TKI resistance. Recent clinical trials with irreversible TKI monotherapy failed to demonstrate an objective response in *EGFR* mutant lung cancer patients that were refractory to treatment with the reversible EGFR-TKIs—gefitinib and erlotinib [34]. These findings suggest that co-existence of altered HGF signaling contributes to the unfavorable response to irreversible EGFR-TKIs in these patients [32]. In addition, the presence of HGF accelerates the growth of pre-existing EGFR-TKI-resistant clones that harbor *MET* amplification [35]. More recently, HGF was reported to induce resistance to ALK selective inhibitors in *EML4-ALK* lung cancer [36] and BRAF inhibitors in *BRAF* mutant melanoma [37]. Such accumulating evidence indicates that HGF is a common target for overcoming resistance to targeted drugs in oncogene-addicted tumors.

Our results also suggest that mTOR inhibitors have advantage of several mechanisms to suppress the growth of EGFR-TKI resistance triggered by HGF in *EGFR* mutant lung cancer cells. For example, these inhibitors likely block mTOR/p70S6K/4E-BP1 survival signals that are usually engaged during PI3K/AKT activation in cancer cells. The PI3K/AKT/mTOR pathway contributes to the survival of *EGFR* mutant lung cancer cells [38], [39]. We previously reported that this pathway is also crucial for acquiring HGF-triggered resistance to EGFR-TKIs in *EGFR* mutant lung cancer cells [40]. In the present study, both temsirolimus and everolimus partially suppressed the phosphorylation of p70S6K and 4E-BP1, as well as inhibited the survival of *EGFR* mutant cancer cells *in vitro*. Thus, mTOR inhibitors achieve growth inhibition by suppressing both survival signals from EGFR and resistance signals from MET in cancer cells.

Another possible mechanism is the inhibition of angiogenesis. Recent studies reported that mTOR-mediated signaling stimulates HIF-1α transcription, which regulates VEGF, via 4E-BP1 phosphorylation [41]. mTOR inhibitors suppress HIF-1α transcription and thereby inhibit VEGF production [28], [29], [41]–[43]. We previously reported that HGF activates the MET/GAB1 pathway and increases VEGF production by *EGFR* mutant lung cancer cells [24]. In the present study, we confirmed this finding and further demonstrated that mTOR inhibitors suppressed both constitutive VEGF production and HGF-stimulated VEGF production. Moreover, we found that while mTOR inhibitors did not affect the viability of unstimulated endothelial cells, they inhibited endothelial proliferation stimulated by various pro-angiogenic factors, including VEGF. mTOR is activated following engagement of several receptor tyrosine kinases such as VEGFR-2, EGFR, and FGFR-1 [41]; hence, mTOR inhibitors may abrogate the stimulation caused by multiple pro-angiogenic factors in endothelial cells. Therefore, mTOR inhibitors may suppress angiogenesis by at least 2 modes of actions. First, they may inhibit VEGF production even in the presence of HGF. Second, they may inhibit endothelial cell proliferation that is stimulated by various pro-angiogenic factors.

Results of recent clinical trials with targeted drugs indicate the importance of patient selection. For example, the response rate of gefitinib was only 10–20% in unselected NSCLC [44], but it was about 80% in *EGFR* mutation-positive NSCLC [43]. The progression-free survival of *EGFR* mutant lung cancer patients was also much longer than that of unselected NSCLC patients [45]. While a large number of mTOR inhibitors have been developed and are being evaluated in clinical trials in lung cancer, neither of sirolimus, temsirolimus, or everolimus, when used as monotherapeutic agents, show clinical efficacy in unselected NSCLC [46] [47]. Moreover, recent studies of mTOR inhibitors combined with EGFR-TKI also failed to show clinical benefit in unselected NSCLC [46] [48]–[50]. Our current study is preclinical, and we demonstrated that mTOR inhibitors showed considerable efficacy in lung cancer cells with HGF-mediated resistance to EGFR-TKI resistance both *in vitro* and *in vivo*. Dong et al. also reported the anti-tumor activity of mTOR inhibitors against EGFR-TKI-resistant *EGFR* mutant lung cancer cells with the T790M gatekeeper mutation [51]. Both HGF and T790M gatekeeper mutations are frequently detected in EGFR-TKI resistant tumors, where they contribute to resistance. Therefore, these observations illustrate the necessity of clinical trials with mTOR inhibitors in *EGFR* mutant lung cancer patients who become refractory to gefitinib and erlotinib. Moreover, since HGF confers resistance to targeted drugs in several tumor types, clinical trials of mTOR inhibitors are warranted.

## Supporting Information

Figure S1 **mTOR inhibitors did not further sensitize** ***EGFR*** **mutant lung cancer cells to erlotinib** ***in vitro*** **.** PC-9/Vec cells were incubated with or without temsirolimus (**A**) or everolimus (**B**), in the presence or absence of HGF (20 ng/ml) and erlotinib (0.3 µM) for 72 h. Then, cell viability was determined by the MTT assay. Bars show SD. The data shown are representative of 3 independent experiments with similar results.
(TIF)

Figure S2 **Knock-down of mTOR inhibited cell growth and VEGF production.** Tumor cells were transfected with mTOR or control siRNA for 24 h and (A) the cell lysates were harvested and subjected to western blotting. (B) cell viability was determined by the MTT assay, (C) VEGF concentration in the supernatants further 48 h after transfection, was determined by ELISA. C shows VEGF levels corrected by the tumor cell number are shown. B and C show quantification of positive cells. *P<0.01, (one-way ANOVA). Bars show SD. The data shown are representative of 5 independent experiments with similar results.
(TIF)

Figure S3 **Temsirolimus did not induce apoptosis in PC-9 cells** ***in vitro*** **, irrespective of the presence of HGF.** PC-9/Vec and PC-9/HGF cells were treated with cisplatin (as a positive control for apoptosis), erlotinib, or temsirolimus for 48 h. Then, the resultant cells were treated with PE Annexin V Apoptosis Detection Kit I.
(TIF)

Figure S4 **Treatment with temsirolimus increased AKT phosphorylation after 4 h treatment, but the phosphorylation was reduced at 96 h.** SCID mice with PC-9/HGF tumors were administered 50 mg/kg temsirolimus. After 4 h, 12 h, 48 h and 96 h, the tumors were harvested, and the relative levels of proteins in the tumor lysates were determined by western blotting.
(TIF)
