# Supplementary figures and images for: mTOR Inhibitors Control the Growth of EGFR Mutant Lung Cancer Even after Acquiring Resistance by HGF
Source: PLoS One. 2013 May 14;8(5):e62104. doi: 10.1371/journal.pone.0062104 (PMC3653905; doi:10.1371/journal.pone.0062104)

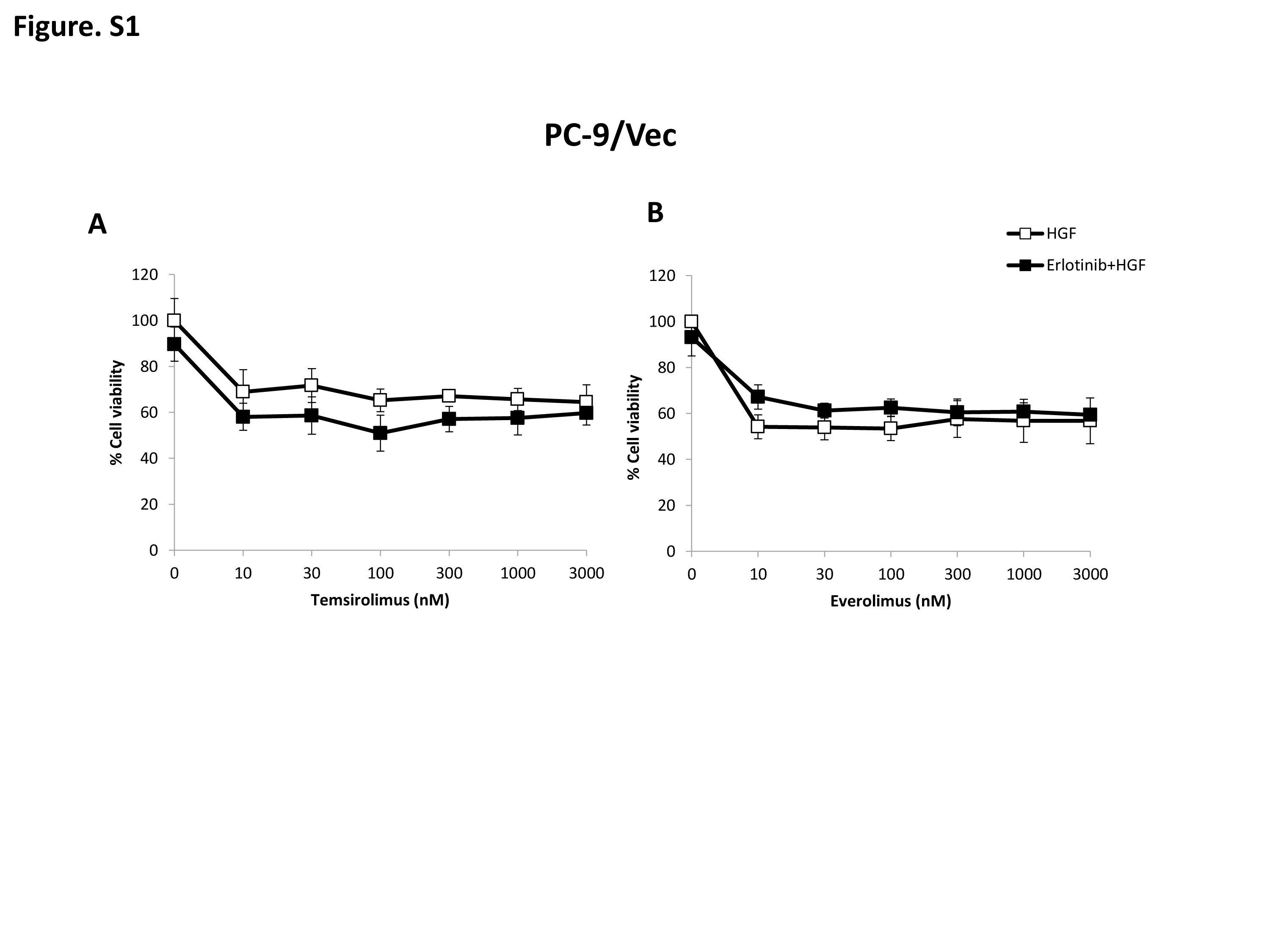

Supplement: Figure S1 — mTOR inhibitors did not further sensitize EGFR mutant lung cancer cells to erlotinib in vitro . PC-9/Vec cells were incubated with or without temsirolimus (A) or everolimus (B), in the presence or absence of HGF (20 ng/ml) and erlotinib (0.3 µM) for 72 h. Then, cell viability was determined by the MTT assay. Bars show SD. The data shown are representative of 3 independent experiments with similar results. (TIF) [file pone.0062104.s001.tif]

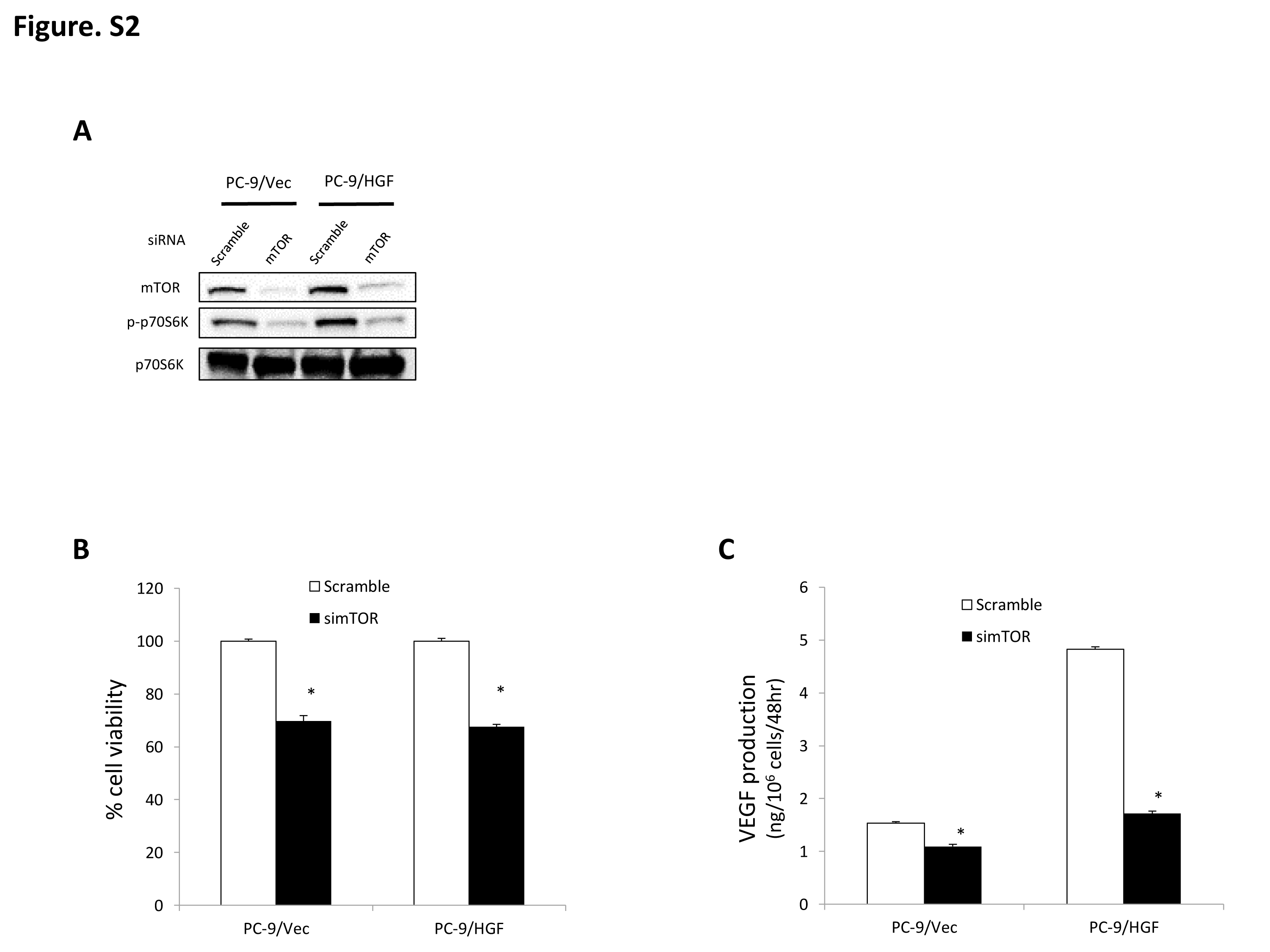

Supplement: Figure S2 — Knock-down of mTOR inhibited cell growth and VEGF production. Tumor cells were transfected with mTOR or control siRNA for 24 h and (A) the cell lysates were harvested and subjected to western blotting. (B) cell viability was determined by the MTT assay, (C) VEGF concentration in the supernatants further 48 h after transfection, was determined by ELISA. C shows VEGF levels corrected by the tumor cell number are shown. B and C show quantification of positive cells. *P<0.01, (one-way ANOVA). Bars show SD. The data shown are representative of 5 independent experiments with similar results. (TIF) [file pone.0062104.s002.tif]

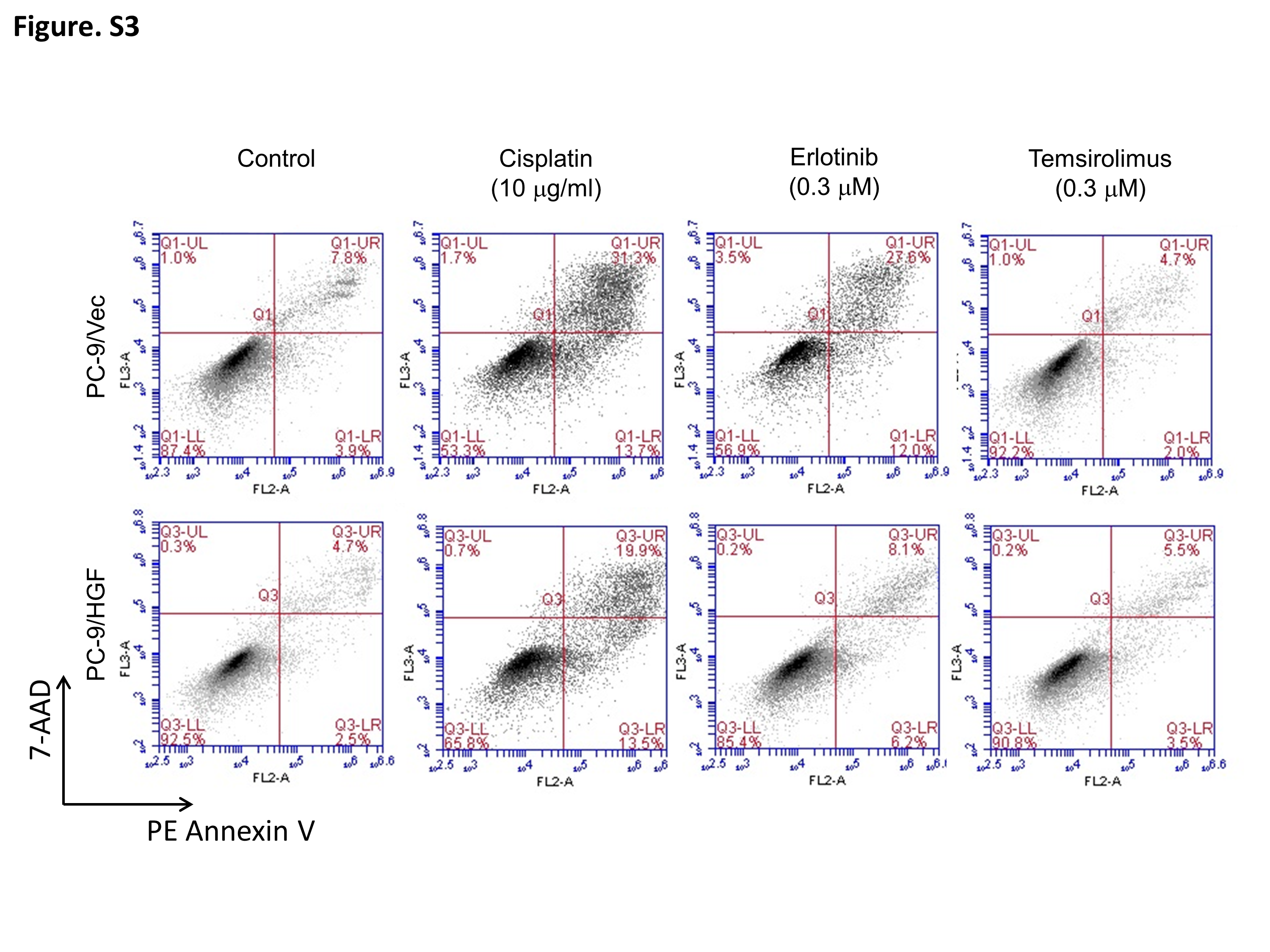

Supplement: Figure S3 — Temsirolimus did not induce apoptosis in PC-9 cells in vitro , irrespective of the presence of HGF. PC-9/Vec and PC-9/HGF cells were treated with cisplatin (as a positive control for apoptosis), erlotinib, or temsirolimus for 48 h. Then, the resultant cells were treated with PE Annexin V Apoptosis Detection Kit I. (TIF) [file pone.0062104.s003.tif]

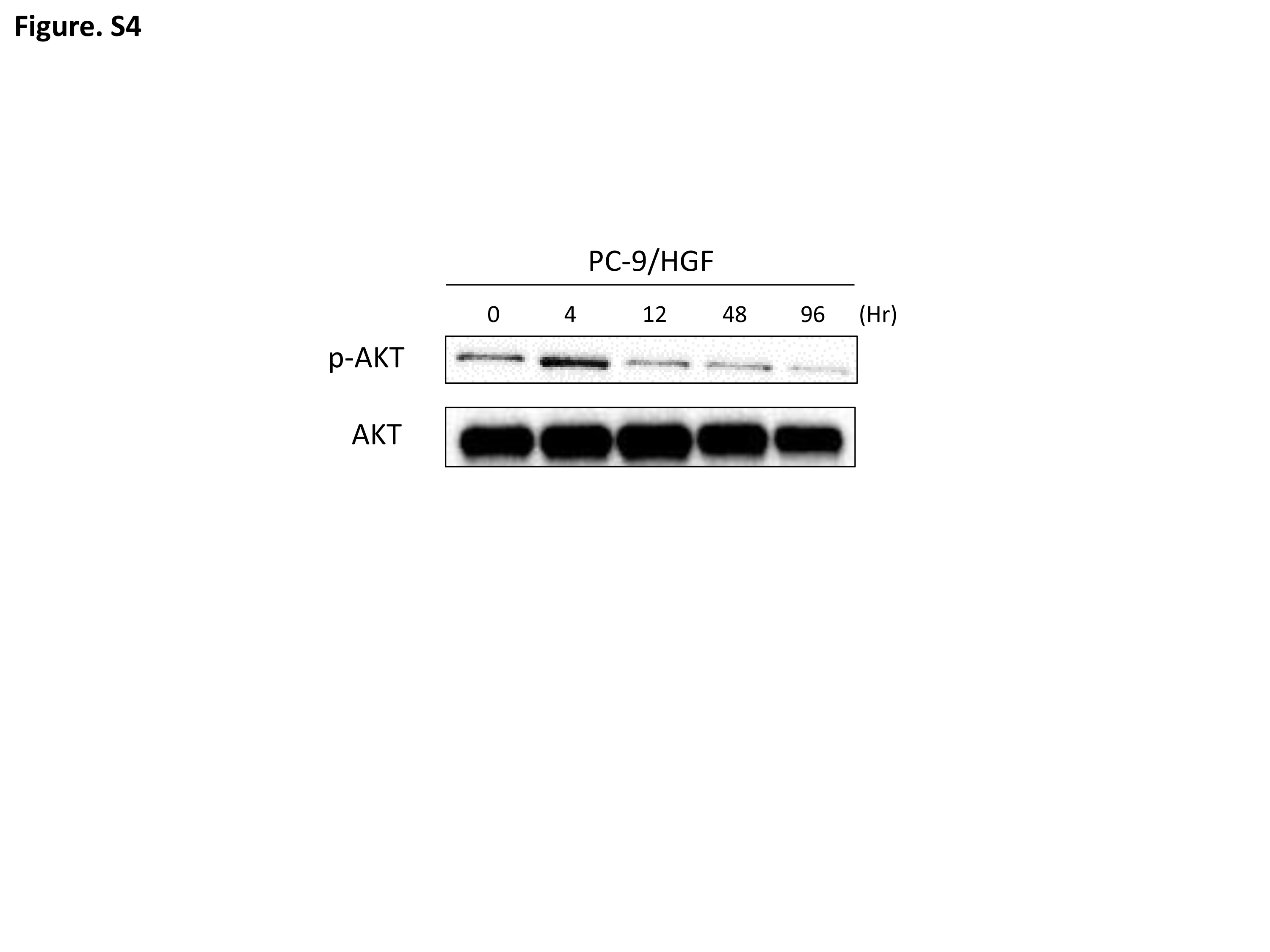

Supplement: Figure S4 — Treatment with temsirolimus increased AKT phosphorylation after 4 h treatment, but the phosphorylation was reduced at 96 h. SCID mice with PC-9/HGF tumors were administered 50 mg/kg temsirolimus. After 4 h, 12 h, 48 h and 96 h, the tumors were harvested, and the relative levels of proteins in the tumor lysates were determined by western blotting. (TIF) [file pone.0062104.s004.tif]
